# A Small Peptide Targeting the Ligand-Induced Androgen Receptor/Filamin a Interaction Inhibits the Invasive Phenotype of Prostate Cancer Cells

**DOI:** 10.3390/cells11010014

**Published:** 2021-12-22

**Authors:** Marzia Di Donato, Pia Giovannelli, Maria Vittoria Barone, Ferdinando Auricchio, Gabriella Castoria, Antimo Migliaccio

**Affiliations:** 1 Department of Precision Medicine, University of Campania “Luigi Vanvitelli”, Via L. De Crecchio 7, 80138 Naples, Italy; marzia.didonato@unicampania.it (M.D.D.); pia.giovannelli@unicampania.it (P.G.); ferdinando.auricchio@unicampania.it (F.A.); 2 Department of Translational Medical Science, University of Naples “Federico II”, Via S. Pansini 5, 80131 Naples, Italy; mariavittoria.barone@unina.it

**Keywords:** prostate cancer, androgen receptor, androgens, cell migration, spheroids

## Abstract

Prostate cancer (PC) is one of the most widespread malignancies among males worldwide. The androgen receptor (AR) plays a major role in prostate cancer development and progression and is the main target of PC therapy. Nonetheless, its action is not yet fully elucidated. We report here that the AR associates with Filamin A (FlnA) promoting migration and invasiveness of various PC-derived cells after androgen challenging. Inhibition of the AR/FlnA complex assembly by a very low concentration of Rh-2025u, an AR-derived peptide specifically interfering with this association, impairs such phenotype in monolayer cells and in 3D models. This study, together with our recent data in cancer-associated fibroblasts (CAFs), indicates that targeting the AR/FlnA complex could improve the clinical management of invasive PC, as the limited number of new drugs reaching the market suggests that we must re-examine the way invasive PC is currently treated. In this context, the synthesis of new biologically active molecules, such as the Rh-2025u peptide, which has been shown to efficiently interfere in the complex assembly in CAFs and PC cells, should overcome the limits of current available therapies, mostly based on hormone antagonists.

## 1. Introduction

The PC development has been since several years hypothesized as a potential consequence of modifications of hormonal environment during male elderly [1]. Therefore, AR has always been regarded as a leading factor in this process and most current therapeutic options for PC still rely on the blockade of AR signaling. It should also be noted that remarkable progress has been made in the PC treatment. Nevertheless, a significant fraction of these cancers progress toward an invasive, metastatic, hormone resistant phenotype with a still poor prognosis. The mechanism controlling prostate cancer (PC) invasion is debated, and even unclear appears the role of the androgen receptor (AR) in this process. As such, therapies for invasive PC still remain unsatisfactory [2].

The most abundant member of the filamin (Fln) family, FlnA binds F-actin through the N-terminus, while it interacts with β1-integrins, GTPase-related proteins, and other partners through its C-terminus. These interactions regulate cytoskeletal remodeling, adhesion, motility and cell spreading [3]. FlnA also interacts with AR to modulate its nuclear translocation and transcriptional activity [4]. We reported that the androgen-bound AR interacts with FlnA, leading to motility, invasion and neuritogenesis in different cell types [5,6,7]. Furthermore, recent findings from our lab have shown that androgen enables the recruitment of cancer-associated fibroblasts (CAFs) by PC cells, allowing a significant increase in PC organoid size in 3D cultures through the remodeling of the extracellular matrix (ECM), which follows the AR/FlnA complex formation [8]. The AR-derived stapled peptide, Rh-2025u, set up in our laboratory, interferes with the AR/FlnA complex and, as a consequence, deranges the cell network surrounding PC organoids, thereby inhibiting the androgen-induced PC organoid growth. This finding suggests that molecules interfering with AR/FlnA could be used to prevent PC progression and also provide a new option to cut down the metastatic potential of PC cells also targeting tumor microenvironment [6,8].

## 2. Materials and Methods

### 2.1. Chemicals and Constructs

Throughout the manuscript, unless otherwise indicated, R1881 and dihydro-testosterone (DHT; both from Sigma-Aldrich, St. Louis, MO, USA) were used at 10 nM, enzalutamide (Selleck-chem, Munich, Germany) was used at 10 μM and the stapled peptide Rh-2025u [6,8], at 10 nM and added 30 min before androgen stimulation. cDNA encoding for the wild-type human AR (hAR) was in pSG5 [9]. The 3416 construct was cloned in the NheI site in pTK-TATA-Luc26 [10]. The plasmid pEGFP-C1 (Addgene; Watertown, MA, USA) was used to generate GFP-stable LNCaP cells [8].

### 2.2. Cell Cultures

LNCaP cells from Cell Bank Interlab Cell Line Collection (ICLC-Genova, Italy), 22Rv1 cells from LGC Standards S.r.L and DuCaP cells [11] obtained from Prof J. A. Schalken were cultured as reported [12]. Seventy-two hours before stimulation, cells were made quiescent as reported [12]. DU145 and PC3 cells from the Cell Bank Interlab Cell Line Collection (ICLC-Genova-Italy) were cultured and made quiescent as reported [13]. Cells were authenticated through DNA profiling by short tandem repeats (STRs) by the supplier. Bromodeoxyuridine (BrdU) incorporation analysis was used for analyzing the cells quiescence and only cells that weakly incorporated BrdU under basal conditions were used. Mycoplasma contamination was routinely monitored. Cell media and supplements were from Gibco (Thermofisher; Waltham, MA, USA). All the cells were manteined at 37 °C in humidified 5% CO_2_ atmosphere.

### 2.3. Transfection, Transactivation and siRNA Experiments

GFP-stable LNCaP cells were generated using the plasmid pEGFP-C1 (Addgene; Watertown, MA, USA) and selected by culture medium containing 1000 µg/mL of G418 (Sigma-Aldrich, St. Louis, MO, USA) [8]. In AR transactivation assay, LNCaP cells were transfected with purified 3416-pTK-TATA-Luc (4 μg) using Superfect Transfection reagent (Quiagen, Hilden, Germany) and left unchallenged or challenged for 18 h with the indicated compounds. The luciferase activity was analyzed as indicated [14]. Lipofectamine 2000 (Invitrogen, Carlsbad, CA, USA) was used in siRNA experiments following the manufacturer’s instructions. For AR siRNA, a pool of four target-specific 19–25 siRNAs (sc-29204; Santa Cruz, Dallas, TX, USA) was used. For Fln A siRNA a pool of three target-specific 19–25 siRNAs (sc-35374; Santa Cruz) was used. Non-targeting siRNA (ctrl siRNA) was from Santa Cruz. Cells were co-transfected with 2 μg of GFP-cDNA (Lonza, Milan, Italy) to identify the transfected cells.

### 2.4. Immunofluorescence (IF), DNA Synthesis, WST-1 and Prostate Specific Antigen (PSA) Assays

To determine cytoskeletal changes, quiescent LNCaP cells on coverslips were unchallenged or challenged with R1881 in absence or presence of enzalutamide and Rh-2025u for 40 min. Cells were fixed with paraformaldehyde (4%, wt/vol in PBS; Merck, Saint Louis, MO, USA), permeabilized with Tween (0.1%, vol/vol in PBS; Biorad, Hercules, CA, USA) and stained with Texas red–labeled phalloidin (Sigma-Aldrich) [8]. Images, representative from at least three different experiments, were generated with DMLB (Leica, Wetzlar, Germany) fluorescent microscope, equipped with HCX PL Apo ×63 oil and HCX PL Fluotar ×100 oil objectives and processed using Leica Suite software (Leica). BrdU incorporation was performed for testing the DNA synthesis. Quiescent cells on coverslips were left unstimulated or stimulated with R1881, in the absence or presence of enzalutamide and Rh-2025u and pulsed with 100 µM BrdU (Sigma-Aldrich, St. Louis, MO, USA) for 18 h as reported [15]. Data were analyzed using a DMLB (Leica; Wetzlar, Germany) fluorescent microscope, equipped with HCX PL Apo ×63 oil and HCX PL Fluotar ×100 oil objectives. The formula: (No. of BrdU-positive cells/No. of total cells) × 100 was used for calculating BrdU incorporation. In AR/FlnA co-localization, AR was revealed using the rabbit polyclonal anti-AR antibody (diluted 1:100 in PBS; sc-815, Santa Cruz Biotechnology, Dallas, TX, USA) and the anti-rabbit fluorescein-conjugated antibody (Jackson Laboratories; diluted 1:200 in PBS containing 0.2% bovine serum albumin). FlnA was detected using the goat polyclonal anti-FlnA antibody (diluted 1:30 in PBS, Ab11074; Abcam, Cambridge, UK) and the rabbit anti-goat Texas red-conjugated antibody (diluted 1:300 in PBS, Abcam). Co-localization was analyzed using a laser scanning confocal microscope [5]. WST-1 reagent (Roche, Basilea, Switzerland) was used to investigate cell proliferation at 24, 48 and 72 h, following the manufacturer’s instructions [13]. The resulting values were expressed as fold increase over the basal level. For PSA analysis, cells were made quiescent and unstimulated or stimulated for 48 h. Conditioned media were collected, and PSA assay was performed using the PSA ELISA kit (Abnova-Taiwan Corporation, Taiwan) [12].

### 2.5. Wound Scratch and Transwell Assays

In wound scratch assay, 1.7 × 10^5^ cells were used in a 24-well plate. Quiescent cells were wounded [16] and left un-stimulated or stimulated for 28 h, in the absence or presence of the indicated compounds. To avoid cell proliferation, cytosine arabinoside (Sigma-Aldrich) at 50 μM (final concentration) was included in the cell medium. Different fields were analyzed using DMIRB inverted microscope (Leica) equipped with N-Plan 10× objective (Leica). Phase-contrast images, representative from three different experiments, were captured using a DFC 450C camera (Leica) and acquired using Leica Suite Software (Leica). The wound width was calculated using Image J Software and expressed as % of the decrease in the wound area. Migration and invasion assays were performed by using collagen- or Matrigel- pre-coated Transwells with 8 μm polycarbonate membrane (Corning; Corning, NY, USA), respectively [16]. The indicated compounds were added and cytosine arabinoside was included in cell medium. Cells were allowed to migrate or invade for seven or 18 h for LNCaP cells respectively and for nine h or 24 h for DuCaP and 22Rv1, respectively. Migrating or invading cells were then scored [16].

### 2.6. Establishment of Spheroids in ECM and Spheroid’s Viability Analysis

LNCaP spheroids were generated [8]. Cells (3 × 10^4^) were mixed in each well with phenol-red free growth factor-reduced Matrigel (10 mg/mL; BD Bioscience) and spheroid plating medium made as reported [13]. After two days, this medium was replaced with a similar medium with the absence of N-acetylcysteine and Y-27632. At the third day, spheroids were untreated or treated as indicated. Different fields were analyzed using a DMIRB Leica (Leica) microscope equipped with C-Plan 40× objective (Leica) and images were acquired using a DFC 450C camera (Leica). The relative spheroid size was calculated [13] and expressed as the fold increase over the basal spheroid size at the third day. At the 15th day, spheroid viability was assessed by 3-(4,5-dimethylthiazol-2-yl)-2,5-diphenyltetrazolium bromide (MTT; Sigma-Aldrich). Matrigel was solubilized with 2% (*w*/*v*) SDS solution. MTT solution (final concentration of 500 μg/mL) was added to spheroids and two hours later, DMSO (100 μL) was added for 1 h at 37 °C. Optical density (O.D.) from triplicate samples was measured at 562 nm using EnSpire plate reader (Perkin-Elmer, Walthman, MA, USA).

### 2.7. Lysates, Immune-Precipitation (IP), Co-Immune-Precipitation (Co-IP), and Western Blot (WB)

Lysates (at 2 mg/mL protein concentration) were prepared as described [13]. The rabbit polyclonal anti-FlnA antibody (sc-28284; Santa Cruz Biotechnology) was used for immunoprecipitating FlnA. AR was revealed using the mouse monoclonal anti-AR antibody (441, sc-7305, Santa Cruz). The rabbit polyclonal anti-FlnA antibody (4762S; Cell Signaling, Danvers, MA, USA) was used to detect FlnA in the immune-complexes and in cell lysates. The mouse monoclonal anti-FAK (610088, BD Transduction Laboratories), anti P-Tyr397 FAK (611722; BD Transduction Laboratories), anti-GAPDH (E-AB-20032; Elabscience, Houston, TX, USA) and anti-Tubulin (T5168; Sigma-Aldrich) antibodies were used for the detection of FAK, P-Tyr397 FAK, GAPDH and tubulin, respectively. Rac pull-down assay was done [8] using the Rac activation kit (Upstate Biotechnology, Burlington, MA, USA). Immune-reactive proteins were revealed by the ECL detection system (GE Healthcare, Chicago, IL, USA).

## 3. Results

### 3.1. Androgen-Increased Growth of LNCaP Cell Spheroid Is Inhibited by the RH-2025u Peptide

**Figure 1:**
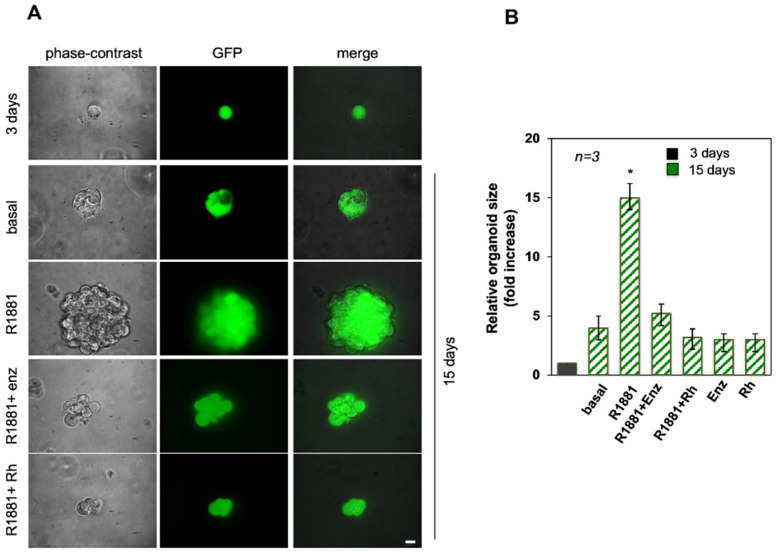
The Rh-2025u peptide effect on the LNCaP-spheroid size. The GFP-LNCaP cells were used. (**A**) After three days, LNCaP in Matrigel were left unchallenged (basal) or challenged as indicated. Peptide or enzalutamide alone were used as controls. (**B**) Representative images by phase-contrast (left section), or fluorescence microscopy (middle section) were captured and shown. Merged images are shown in the right section. Scale bar, 100 μm. In (**B**), the spheroid size was monitored for 15 days and calculated under basal conditions (three days) or in cells left unstimulated (basal) or stimulated for 15 days, in the absence or presence of the indicated compounds. It was expressed as fold increase in the relative organoid size. *n* represents the number of experiments. Means and SEM are shown. * *p*  <  0.05, for the indicated experimental point (R1881) versus the corresponding untreated control (basal).

3D cell cultures provide enhanced models for the testing of drug delivery compared to conventional 2D cultures due to their more physiological cell-cell contact geometry, and mechanical properties. This technique was used to investigate a classical PC model, the LNCaP cells. The ability of the cells to form spheroids in 3D models under hormone stimulation was assayed. GFP-LNCaP cells were obtained [8] and plated in Matrigel for allowing the formation of spheroids after three days of culture, as shown in Figure 1A. On the third day of culture, the spheroids were left unchallenged (basal) or challenged with 10 nM R1881 in the absence or presence of 10 µM of the AR specific antagonist enzalutamide or 10 nM Rh-2025u. The Rh-2025u peptide derives from the AR 622–670 amino acid sequence, critical for interaction of the receptor with FlnA [5]. The stapled peptide was used for its enhanced cell permeability, stronger affinity and decreased degradation as compared to its un-stapled counterparts. The cell medium was changed every three days and the spheroid size was monitored for 15 days. Representative images (Figure 1, panel A) by phase-contrast (left section), or fluorescence microscopy (middle section; GFP) were captured. The corresponding merged images are shown in the right section (Figure 1, panel A). Quantification of data (Figure 1, panel B) shows that after 15 days, the spheroid size was increased by less than fivefold when the cells were untreated. In contrast, androgen stimulation increased by about the sizes of the spheroids by a factor of about fifteen. As expected, the enzalutamide significantly (*p* < 0.05) reduced the hormone effect and, interestingly, an even stronger inhibition was observed when the spheroids were treated with R1881 in the presence of the peptide Rh-2025u, which disrupts the AR/FlnA association. Figure 1, panel B shows that the enzalutamide and Rh-2025u used in the absence of hormone have no effects on spheroid growth. These findings lead to the conclusion that the androgen stimulates the size increase of PC cells derived spheroids, and that this action is likely mediated by the AR/FlnA complex, as it is abolished by the peptide interfering with the assembly of this complex. 

### 3.2. The Peptide Treatment Does Not Affect LNCaP Cell Proliferation in 2D and 3D Models Nor AR Induced Transcriptional Activity

**Figure 2:**
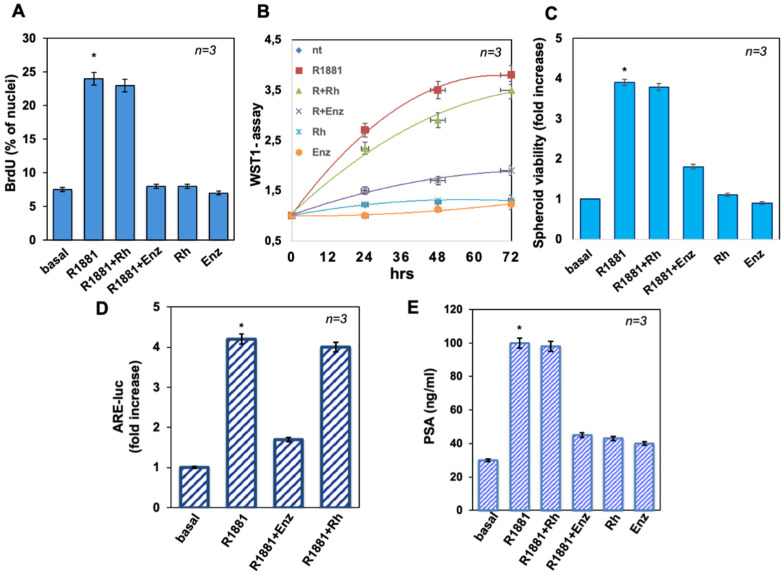
The Rh-2025u peptide does not affect LNCaP cells proliferation and viability. Quiescent LNCaP cells were employed. (**A**) Cells were left unchallenged or challenged for 18 h with the indicated compounds and pulsed in vivo with 100 μM BrdU, then analyzed by immunofluorescence (IF) and expressed as the percentage of nuclei. (**B**) Cells were left untreated or treated for 24, 48, and 72 h with the indicated compounds. WST-1 reagent was used for testing cell proliferation and graph represents the ratios of proliferation expressed as the fold increase over the basal absorbance. R1881 induced a significant (*p* < 0.05) increase in cell proliferation as compared with the untreated (nt) cells. (**C**) LNCaP-Spheroid viability was analyzed by the MTT assay after 15 days of treatment. Graph represents the ratios of proliferation expressed as the fold increase over the basal absorbance. (**D**) Cells were transfected as reported in Methods. Luciferase activity was assayed, normalized using β-gal and expressed as fold induction. (**E**) Cells were left unstimulated or stimulated for 48 h with R1881 in absence or presence of the indicated compounds and conditioned media were collected and analyzed by ELISA. The graph represents the release of PSA expressed in ng/mL and detected by absorbance. In (**A**–**E**), *n* represents the number of experiments. Means and SEM are shown. When indicated, * *p*  <  0.05, for the indicated experimental point (R1881) versus the corresponding untreated control (basal). In (**A**–**E**) only the enzalutamide inhibits significantly the effects triggered by R1881.

To evaluate the mitogenic effect of androgen in 2D and 3D cultures, BrdU incorporation and proliferation were assayed. R1881 was stimulated by about threefold the number of cells incorporating BrdU, as compared to unstimulated (basal) cells. While enzalutamide completely impaired the R1881-elicited effect, the Rh-2025u peptide had no effect on the DNA synthesis stimulated by the androgen. Enzalutamide and the stapled peptide did not significantly affect the BrdU incorporation when used alone, as controls (Figure 2A). Similar data were obtained using 10 nM DHT (Figure S1A). WST-1 assay was performed for evaluating the effect of R1881 on cell proliferation. R1881 treatment stimulated the proliferation of LNCaP cells, with an effect already evident after 24 h. Also in this case, enzalutamide inhibited the androgen-elicited effect, which persisted until 72 h of treatment, while the stapled peptide did not significantly affect the R1881-induced proliferation. Here again, enzalutamide and the stapled peptide left unaltered the cell proliferation, when used alone, as controls (Figure 2B).

The spheroid’s viability was also analyzed by 3-(4,5-dimethylthiazol-2-yl)-2,5-diphenyltetrazolium bromide (MTT) assay. Figure 2C shows that R1881 increased by about fourfold the viability of spheroids from LNCaP cells. Enzalutamide decreased this effect, while Rh-2025u did not perturb it. Both the compounds did not modify the spheroid viability, when added alone, as controls. This finding strongly suggests that the increase in the spheroid size is not the product of an increase of cells number but this effect is due to rearrangements of the cell network built up following the interaction between AR and Filamin A. 

The effect of the AR/FlnA interaction was also probed on hormone induced gene transcription (Figure 2D). To this end, LNCaP cells were transfected with the 3416 ARE-luc construct and left unchallenged (basal) or challenged for 18 h with 10 nM R1881, alone or in the presence of 10 µM Enzalutamide or 10 nM Rh-2025u. Luciferase activity was assayed [8] and expressed as fold increase. As expected, R1881 strongly stimulates reporter gene transcription. Enzalutamide, but not the Rh-2025u peptide, completely suppressed this stimulation, confirming that the peptide only affects the AR/Fln A interaction, leaving unchanged the hormone action on gene transcription. Consistent with this finding, panel E in Figure 2 shows that the antagonist, but not by the peptide, inhibited the androgen-stimulated PSA secretion by LNCaP cells. Neither enzalutamide nor the Rh 2025u peptide had any effect on PSA basal level. 

Collectively these findings implicate that the AR/FlnA interaction is not involved in hormone stimulated cell growth in both 2D and 3D models nor affects transcriptional activity of AR. Therefore, it can be hypothesized that the observed spheroid outgrowth is a consequence of the functional interaction of the AR and FlnA, which drives cell motility by exquisitely non-transcriptional mechanisms.

### 3.3. The Rh-2025u Abolishes the Androgen Stimulated Motility and Invasion of LNCaP Cells

Our previous findings in different cell types raised the question as to whether the androgen-induced AR/FlnA complex stimulates motility and invasion of epithelial PC cells.

**Figure 3:**
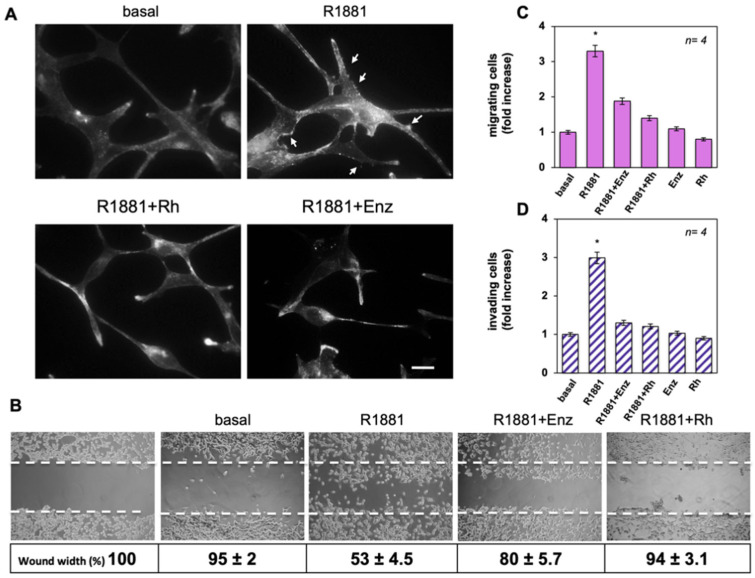
The Rh-2025u impairs the migratory behavior in LNCaP cells. Quiescent LNCaP cells were used. (**A**) Cells, unstimulated or stimulated for 40 min as indicated, were analyzed for F-actin and arrows indicate cells protrusions and ruffles. The Immunofluorescence images (IF) are representative of 3 different experiments, each in duplicate. Scale bar 10 μm. In (**B**) cells were wounded and unstimulated or stimulated as indicated. Phase-contrast images are representative of three different experiments, each in triplicate. The wound area was calculated and data, expressed as the percentage of decrease in wound width over the control cells (analyzed at time 0; left panel), are reported below each image. In (**C**,**D**), cells unchallenged or challenged as indicated, were allowed to migrate (**C**) or invade (**D**) for seven h or 24 h, respectively and scored as described in “Methods” section. Means and SEMs are shown. In (**C**,**D**), *n* represents the number of experiments. * *p*  <  0.05 for the indicated experimental points versus the corresponding untreated control (basal).

As modifications of the cytoskeleton are required for cell migration, we firstly analyzed by IF microscopy the effect of androgens on actin remodeling in LNCaP cells. Figure 3A shows that 10 nM R1881 treatment triggered the formation of ruffles and protrusions (upper panels) within 40 min. Addition of enzalutamide or Rh-2025u reverted such effect. The appearance of spikes induced by androgen is consistent with an increase of cell locomotion.

We assessed the influence of androgens on motility and invasion of LNCaP cells. Firstly, cells were wounded and left to migrate in the absence or presence of the indicated compounds. Findings from the wound healing assay shown in Figure 3B are even more impressive: here it can be noted that a significant number of cells migrated in the wound area upon androgen treatment, while enzalutamide and more strongly the stapled peptide avoided the R1881-induced effect. Images captured at 0 time or from untreated cells (basal) were also acquired and presented for comparison (Figure 3B). Shown below the images are the corresponding percentage of wound width reduction. They indicate that the wound width was significantly (*p* < 0.05) diminished in cells treated with R1881, as compared with control basal cells. Enzalutamide and Rh-2025u reverted the effect elicited by R1881, albeit at different extent, while exhibiting a negligible effect when used in the absence of R1881. 

Thus, we studied the R1881 effect on migration and invasiveness of LNCaP cells by using collagen- and matrigel-coated transwells. R1881 increased by ~3.2- and 2.9-fold the number of migrating (Figure 3C) or invading (Figure 3D) cells, respectively. Enzalutamide or Rh-2025u peptide inhibit both the androgen effects, while leaving almost unaffected migration or invasion of cells, when used in the absence of hormone. Also in this case, 10 nM peptide works better than 10 µM enzalutamide in inhibiting migration induced by R1881 in LNCaP cells. Similar data were obtained on cell migration using DHT (Figure S1B).

The impact of hormone on cell motility and invasion was also analyzed using increasing concentrations from 0.1 to 10 nM of R1881. The results in Figure S1C,D show that R1881 stimulates both cell motility and invasion in a concentration dependent fashion. Notably, the peptide (at 10 nM) inhibits the androgen-stimulated effects, regardless of hormone concentration. Thus, its inhibitory effect on both migration and invasion strongly suggests that hormone action on cell motility relies on the formation of the AR/FlnA complex in PC cells.

### 3.4. The Rh-2025u Abolishes the Androgen Stimulated Motility and Invasion of AR Expressing Cells but Not of PC Cells That Do Not Express AR

**Figure 4:**
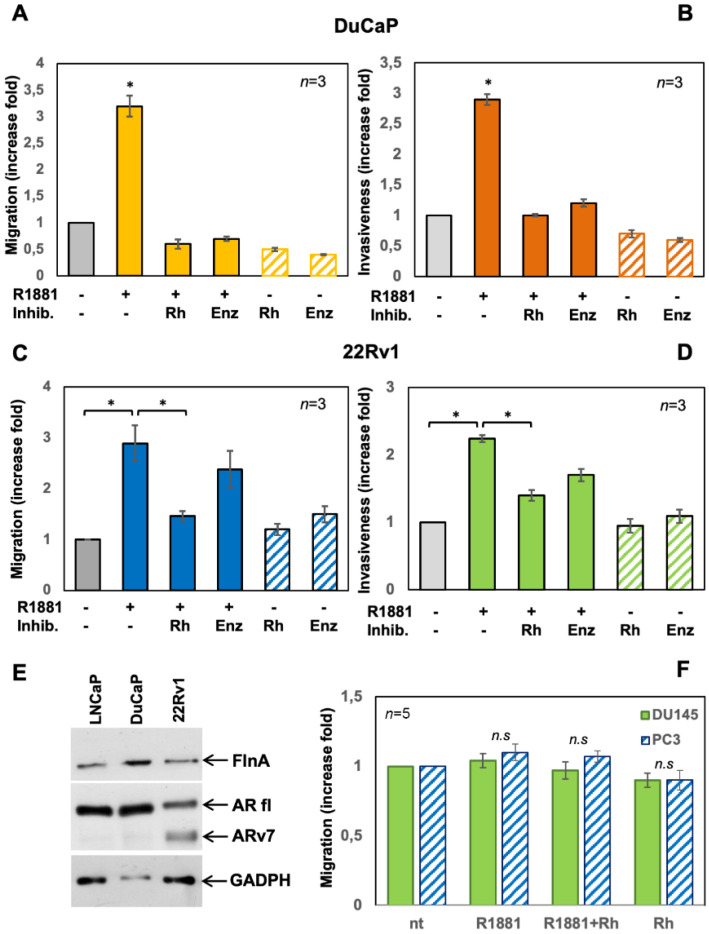
The inhibitory effect of Rh-2025u on androgen-induced migration and invasiveness of various PC cells. Quiescent DuCaP (**A**,**B**), 22Rv1 (**C**,**D**), DU145 and PC3 (**F**) cells were used. In (**A**–**F**), cells, left unstimulated or stimulated as indicated and allowed to migrate (**A**,**C**,**F**) or invade (**B**,**D**) for nine h or 24 h, respectively, were scored as described in “Methods” and data were expressed as increase fold. Means and SEMs are shown. *n* represents the number of experiments. * *p*  <  0.05 for the indicated experimental points versus the corresponding untreated control (basal). In (**C**,**D**), only the Rh-2025u inhibits significantly (*) the cell migration (**C**) and invasiveness (**D**) triggered by androgens. In (**E**), lysates from the indicated cell lines were prepared and analyzed by Western blotting using the antibodies against the indicated proteins. AR fl stands for the AR full length isoform. ARv7 for the AR truncated form.

To better assess the specificity of the peptide action, we analyzed its effect in different PC cell lines, which express different amounts and forms of AR. Thus, the AR expressing cell lines DuCaP and 22Rv1 cells were selected. DuCaP cells were derived from a metastatic lesion to the dura mater of a patient with hormone refractory PC. These cells express considerable amounts of wild type, full length AR [17]. They were challenged with 10 nM R1881 in the absence or presence of 10 nM Rh-2025u peptide or 10 µM enzalutamide. As in LNCaP cells, the hormone strongly stimulated both migration and invasiveness of DuCaP cells and the peptide as well as enzalutamide completely eliminated this effect. Both peptide and enzalutamide had no effect when added in the absence of hormone to cell medium (Figure 4A,B). We then analyzed the effect of the androgen in 22Rv1 cells, a human PC cell line derived from a xenograft that was serially propagated in mice after castration-induced regression and relapse of the parental, androgen-dependent CWR22 xenograft. In addition, to express the full-length AR, these cells exhibit a truncated form of the receptor, named ARv7, lacking the whole hormone-binding domain at carboxy-moiety of the protein [18,19]. ARv7 splicing variant is expressed less than 1% in primary PC but is detected in ~75% of cases in patients affected by PC following ADT protocols and further its expression increases in response to abiraterone acetate or enzalutamide therapy. In CRPC, ARv7 is predominantly localized in the nuclei of the cells and its expression correlates with that of AR-FL [18]. Therefore, ARv7 is considered as a marker of hormone-refractoriness and has been hypothesized to be involved in the development of castrate-resistant PC phenotype. The findings obtained in 22Rv1 cells look particularly interesting. The androgen stimulated migration and invasiveness of 22Rv1 cells and only the peptide efficiently inhibited the hormone stimulated cell migration, whereas enzalutamide did not significantly interfere with the hormone action (Figure 4C). Both, Rh peptide and Enzalutamide only partially inhibited the androgen-induced cell invasiveness, however Rh-2025u has a stronger inhibitory effect than enzalutamide (Figure 4D). Also, in 22Rv1, both Rh-2025u peptide and enzalutamide had no effect on cell migration/invasion in the absence of androgen (Figure 4A,B). The results observed in 22Rv1 cells could be explained by hypothesizing that both full-length AR and ARv7 contribute, likely by dimerization, to mediate the hormone-induced cell migration. Therefore, the hormone antagonist enzalutamide fails to inhibit cell motility sustained by the truncated form of the receptor (ARv7), which is unable to bind ligands, whereas Rh-2025u peptide acting downstream in the receptor signaling efficiently suppresses the hormone action. The Fig. 4E shows the expression of Filamin A, full length and AR7 androgen receptor expression in LNCaP, DuCaP and 22Rv1 cells.

Finally, data in Figure 4F indicate once more that the stapled peptide is AR-specific, since the hormone does not stimulate cell migration and Rh-2025u does not work in AR-negative PC-3 and DU145 cells.

**Figure 5:**
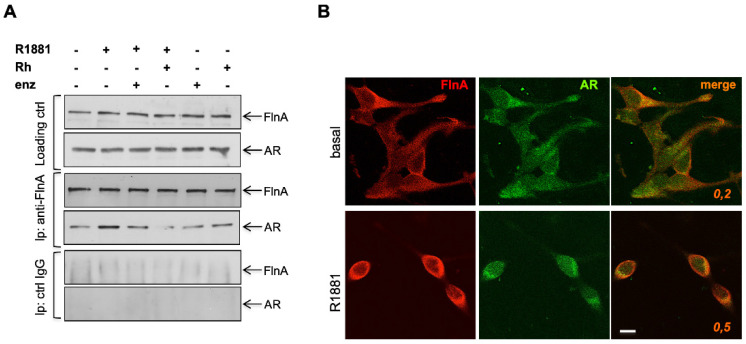
The androgen triggers AR/FlnA complex assembly and co-localization. Quiescent LNCaP were used. In (**A**), cells were left unchallenged or challenged for 10 min as indicated. The upper section shows the WB with the indicated antibodies to reveal lysate proteins (loading), which were then immunoprecipitated using the anti-FlnA (filamin A) antibody (middle section) or control IgG (lower section, ctrl IgG). Results are representative of three different experiments. In (**B**), cells were stained for AR and FlnA. Images captured by confocal microscope, and representative of three independent experiments, show the staining of Fln A (red) and AR (green). On the right merged images are presented. Bar, 5 μm. Co-localization factors are indicated in the right panels.

This conclusion is supported by the findings from pull-down experiments presented in Figure 5A, showing that R1881 increases the specific co-immunoprecipitation of AR with FlnA, showing that a physical interaction exists between these two proteins. Consistent with these data, R1881 increases by 2.5-fold the co-localization ratio between AR and FlnA in the extra-nuclear compartment of cells, as shown by confocal microscopy (Figure 5B). The Rh-2025u peptide reverses the complex assembly, with an efficacy even stronger than that observed upon enzalutamide treatment and such a difference is coherent with the more robust inhibition exerted by the peptide in invasion and motility assays, as well as in 3D models.

### 3.5. Filamin A and AR Are Both Required for LNCaP Cell Motility Induction by a Mechanism Involving Rac Activation and Fak Phosphorylation

**Figure 6:**
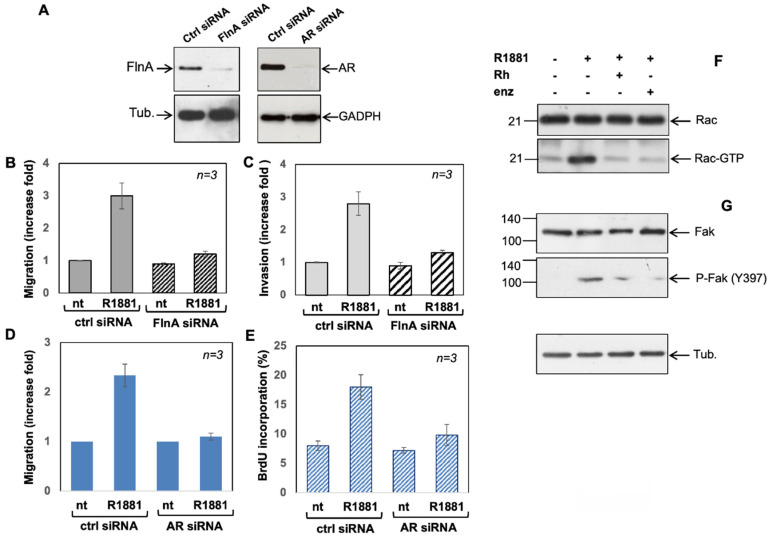
The expression of Filamin A and the activation of Rac 1 and FAK (Tyr 397) are important for androgen induced LNCaP migration. In (**A**–**E**), siRNA Alexa Fluor 488, control siRNA (Ctrl siRNA) or siRNA FlnA (FlnA siRNA) or siRNA AR (AR siRNA) were used as in “Methods” section. (**A**) After transfection, cellular lysates were obtained and FlnA (left) and AR (right) expression were analyzed by WB using the appropriate antibodies. Transfected and quiescent cells, unstimulated or stimulated as indicated, were used for migration (**B**–**D**) or invasiveness assays (**C**–**E**) for seven h or 24 h, respectively. Migrating (**B**–**D**) or invading (**C**–**E**) cells were scored as in “Methods” section and data expressed as fold increase. Means and SEM are shown. In (**F**), proteins lysates were used for Rac pull down assay using a commercially available kit, as described under “Methods”. The WB with anti-Rac antibody revealed the total amount of Rac expressed in the corresponding lysates (upper panel) and the eluted Rac (Rac-GTP; lower panel). In (**G**), lysate proteins were analyzed for Fak activation (P-Fak Y397), using the anti-P-Tyr397Fak antibody. The filter was stripped and re-probed using anti-Fak antibody (upper section). The WB for tubulin expression in lysate proteins was finally done, as loading control. In (**A**–**E**) results are representative of 3 different experiments and when indicated *n* represents the number of experiments. In (**B**–**E**), in ctrl siRNA transfected cells, R1881 increases significantly the cell migration (**B**,**D**) or BrdU incorporation (**C**,**E**).

The role for both AR and Filamin A in promoting PC cell migration and invasion was then analyzed in the knockdown experiments shown in Figure 6A–E. Figure 6A shows that FlnA and AR were silenced in FlnA siRNA (left) and AR siRNA (right) transfected cells, compared with control cells, transfected with non-targeting siRNA (Ctrl siRNA). Silencing of FlnA significantly (*p*  <  0.05) reduced the amount of migrating (Figure 6B) or invading (Figure 6C) cells upon androgen stimulation. Noteworthy, transfection of cells with non-targeting, control siRNA did not eliminate the androgen stimulatory effects. Similarly, silencing of AR both abolished the androgen-triggered motility (Figure 6D) and invasiveness (panel Figure 6E) of LNCaP cells stimulated by the hormone.

To get insight into the molecular mechanism by which the AR/FlnA complex promotes the motility/invasiveness of LNCaP cells, we looked at two main effectors of cell motility, the small G-protein Rac and the focal adhesion kinase (Fak). Figure 6F clearly shows that androgen induces a sharp increase of Rac active form (Rac-GTP), which is abolished by addition of enzalutamide or Rh 2025u peptide. Analogously, the hormone stimulates Fak activation (Figure 6G), and this effect is almost completely abolished by both enzalutamide and the peptide. Overall, these findings show that the androgen stimulates cell motility of LNCaP prostate cancer cells forming a complex with Filamin A, which, in turn, stimulates Rac and Fak activation.

## 4. Discussion

Significant advances have been made in the management of PC, which represents one of the most common forms of cancer in males. Different risk factors have been associated with PC, including age, race, heredity, and obesity [20]. Tumor suppressors, oncogenes, and polymorphisms have been and are even now being analyzed. Clinical data suggest that this tumor is generally well controlled as long as it remains in situ. Mortality from PC is dramatically increased upon the spreading of cancer cells from the primary organ to form metastatic tumors at distant sites. Actually, PC is very hard to be managed once it has metastasized. Therefore, the motility gain of PC cells represents one of the main issues related to PC progression and taking appropriate action in the early stages of the disease before it progresses is a major medical priority. AR remains a driving force in the development and progression of PC.

At early stages the management protocol of patients is represented by surgery or radiotherapy, which fail in 10–20% of cases. Later, recurrent patients are subjected to androgen deprivation therapy (ADT), whose efficacy is time-limited so that most patients develop castration-resistant prostate cancer (CRPC) [21,22]. This is a multifaced type of cancer, in which often cells still depend on AR for migrating or growing in presence of very low levels of circulating androgens [23]. Unfortunately, mutations affecting the receptor (gain-of-function, amplification/over-expression, androgen independent AR activation, splice variants) cause the treatment failure [23,24,25]. In this scenario, the development of new drugs targeting AR and its direct interactors for blocking migration of PC cells represents a critical need.

Several proteins have been called into question, such as inflammatory cytokines like IL-1ß and cyclo-oxygenase 2 (COX-2), or heat shock protein 27 (HSP27) whose overexpression appears to be correlated with PC cells invasion and metastasis [26,27]. Nonetheless, the mechanism underlying the role of HSP27 in driving PC cells migration from the prostate to distant metastatic sites is still elusive [27]. Conversely, PSA, acting as a transient receptor potential melastatin 8 (TRPM8) agonist, has been shown to reduce motility of the AR-negative PC3 cell line, suggesting that plasma membrane TRPM8 receptor could exert a protective role against PC progression [28]. In this context, more recently, new selective TRPM8 antagonists have emerged as potential drugs for inhibiting both the androgen-induced proliferative and migratory behavior of various AR-expressing PC cells and the spheroids size-growth in extracellular matrix (ECM) through the modulation of non-genomic actions ([12]). PC invasion is also related to the cadherin-catenin adhesion system, critical for the maintenance of normal tissue architecture and regulated by a family of proteins collectively named cell adhesion molecules (CAMs). Abnormal expression of N-cadherin has been found in different cancers including PC [29]. In this regard, the role for the metalloproteases (MMPs) is also noteworthy. High expression of ADAM-15 [30] and ADAM-12 [31] were found in PC tissues and sera/urine of patients affected by PC with advanced pathological tumor stages with metastasis, respectively. MMP-9, positively correlated to nerve growth factor (NGF) and glial cell-derived neurotrophic factor (GDNF) in PC patients with lymph-vascular, extra-prostatic and perineuronal cell invasion, suggesting that the interactions between NGF, GDNF and MMP-9 during the transition to malignancy may play a role in PC aggressiveness [32]. This seems consistent with the finding that alterations in the expression of neurotrophic factors and their receptors (Trk tyrosine kinases and p75^NTR^) occurs during prostate carcinogenesis [33]. Targeting NGF–TrkA signaling in PC could also directly affect the signaling pathways involved in PC cells migration [13,34].

Specific genes have been involved in the regulation of metastatic potential of PC cells. Experimental and clinical evidence suggests that N-myc downregulated gene 1 (NDRG1) acts as a suppressor of PC metastasis. Although the molecular mechanisms that lead NDRG1-deficient PC cells to increased invasiveness remain largely unknown, it has been shown that NDRG1-deficient prostate tumors have decreased integrin expression and reduced cell adhesion and motility, likely due to downregulation of active RhoA and Rac1 GTPases, as well the concurrent upregulation of active Cdc42 [35]. In addition, an increasing number of miRNAs has been involved in PC cell motility. For example miR-21, regulating the expression of proteins relevant to cell kinesis, including PDCD4, TPM1, and MARCKS, promotes motility, and invasion in PC cells [36]. Conversely, miR-124 and miR-133 exert an inhibitory effect on PC cell migration by targeting talin 1 [36] and EGFR, respectively [37]. Many of these studies were carried out in hormone independent cells, as the metastatic process is generally associated with the hormone-independent phenotype.

Several reports over the years pointed to a role of Src/Fak in PC cell motility. It has been shown that CXCL13 induces CXCR5-dependent activation of the PI3K-p85α in LNCaP cells, and p85α as well as -p101 in PC3 cells. CXCL13-CXCR5 interaction regulates LNCaP and PC3 cell migration and invasion through ERK1/2 kinase activation dependent on the PI3K-p110 isoform(s), Src, and Fak [38]. TheCX3CL1/C-X3-C motif chemokine receptor 1 (CX3CR1), which is overexpressed in PC tissues with spinal metastasis, appears to promote cell migration by activating the Src/Fak pathway, as inhibitors of these kinases suppressed the cell migration induced by CX3CL1 or CX3CR1 overexpression [39]. On the other hand, HCRP-1 depletion, which induced lung metastasis of PC cells in xenograft model, triggers Src and Fak phosphorylation [40]. Surprisingly, the role of androgens, which play a pivotal role in the early phases of tumor development, is still debated as regards cell migration and invasion [41]. Androgens have been demonstrated to activate the Src/Ras/ERK pathway in PC cells [42] and the activation of this cascade is required for hormone induced DNA synthesis. This pathway has been also involved in androgen induced migration of triple negative breast cancer cells [16] but not in PC cells, where the Fak phosphorylation, the activation of cell motility and invasion appear to be strictly dependent on the formation of the AR/FlnA complex. Noteworthy, the AR/FlnA complex, recruiting integrin β1, forms a ternary complex that controls focal adhesion kinase (Fak), paxillin and Rac, thereby driving migration of prostate CAFs [8]. Furthermore, it has been observed that in mesenchymal cells, upon association with membrane type-matrix metalloproteinase 1, this complex activates a protease cascade triggering extracellular matrix remodeling in prostate CAFs [8]. These findings indicate that, depending on cellular environment, androgens exploit separate and specific pathways to promote DNA synthesis and proliferation or cell motility, and this has noticeable implications in PC biology. In fact, it is known that most of prostate cancers, including those apparently independent on hormone for growth, express significant amounts of AR. It is conceivable that, the androgens act on PC as a whole, meaning they can work collectively on its parenchymal and mesenchymal constituents, and even though have negligible effects on cell growth, could retain a crucial role in the control of the tumor reshaping and metastasizing.

The availability of a specific inhibitor of the AR/FlnA complex acting downstream of hormone binding could provide a second line option to repress the metastatic potential of hormone refractory cancers bearing constitutively active AR. Very intriguingly, the findings in 22Rv1 cells, bearing both wild type AR and a truncated AR form (ARv7) lacking the hormone binding domain, show that androgen is still able to stimulate cell motility and invasion, but enzalutamide has negligible effects on the hormone action whereas the peptide completely inhibits the hormone-induced motility/invasion. This observation is consistent with the notion that expression of splice variants such as ARv7 lacking the ligand binding domain (LBD) and exhibiting a constitutive transcriptional activity can be responsible for reactivation of AR activity after androgen deprivation therapy in castration-resistant prostate cancer (CRPC). ARv7 is indeed considered a marker of resistance to 2nd generation androgen receptor signaling inhibitors such as abiraterone acetate and enzalutamide but could also be a driver of lethal resistance via its ligand-independent activity [43]. These data are also supported by two ongoing clinical trials focused on the ARv7 (NCT03236688; NCT03103724). While the EXCALIBUR study (NCT03103724) has been recently completed but data are not available, the NCT03236688 trial is still active, and is focused on identifying the ARv7 splice variant transcripts in exosomes derived by blood circulation of metastatic CRPC patients pre- and post-treatment with selective androgen pathway inhibitors (i.e., abiraterone and enzalutamide). In the complexities of cell-to-cell communication, exosomes emerge as “riders” acting as biological material exchangers between epithelial and stromal cells or as “hackers” of the dormant cells in PC tumor microenvironment [44]. In this scenario, they may also have many potentialities for drug delivery or as non-invasive diagnostic tools in PC [45].

The findings now presented suggest that Rh-2025 peptide could overcome the resistance to the 2nd generation inhibitors, by interfering with the downstream signaling of AR/FlnA complex.

In conclusion, the current research aims to discover many targets which could be “druggable” for inhibiting invasiveness of PC cells. The stapled peptide here used could be placed in this area, but with an added value. By inhibiting the invasiveness of both PC and its surrounding cells, it stands as an excellent candidate in this field. By this approach, PC epithelial cells and their microenvironment could be evaluated and targeted not as two distinguished compartments, but as one. These findings try to address the need for a new generation of drugs for metastatic PC therapy.

## Data Availability

Not applicable.

## Supplementary Materials

The following are available online at https://www.mdpi.com/article/10.3390/cells11010014/s1, Figure S1: In (A–D), quiescent LNCaP cells were used. When indicated, Dihydrotestosterone (DHT) was used at 10 nM, enzalutamide (Enz) at 10 μM and Rh-2025u (Rh) at 10 nM.
