# Supplementary material for: A Small Peptide Targeting the Ligand-Induced Androgen Receptor/Filamin a Interaction Inhibits the Invasive Phenotype of Prostate Cancer Cells
Source: Cells. 2021 Dec 22;11(1):14. doi: 10.3390/cells11010014 (PMC8750472; doi:10.3390/cells11010014)
Supplement: Supplementary file 1 [file cells-11-00014-s001.zip › cells-1451798-supplementary.pdf]

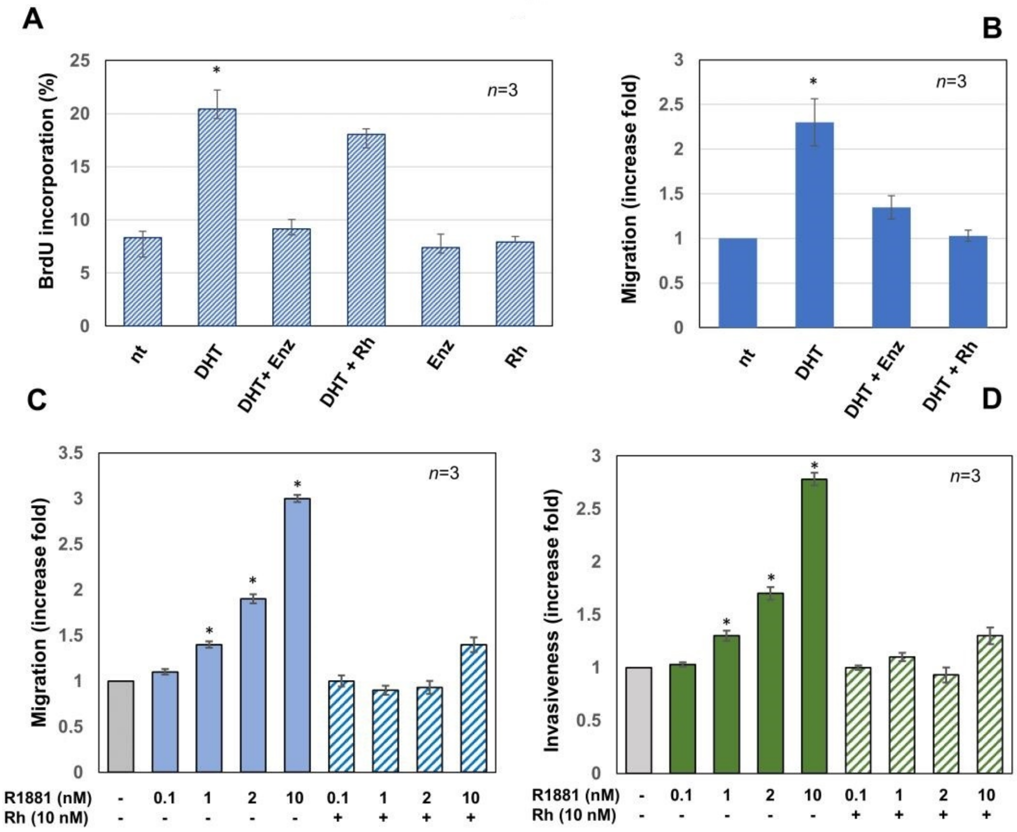

Figure S1. In A-D, quiescent LNCaP cells were used. When indicated, Dihydrotestosterone (DHT) was used at 10 nM, enzalutamide (Enz) at 10 µM and Rh-2025u (Rh) at 10 nM. A) Cells were left untreated or treated for 18 h with the indicated compounds and pulsed in vivo with 100 µM BrdU. Its incorporation into DNA was analyzed by immunofluorescence (IF) and expressed as the percentage of nuclei (%). B) Cells were left unchallenged or challenged with DHT, in the absence or presence of the indicated compounds, allowed to migrate for 7 h in Boyden's chambers and scored as described in "Methods" section. Data were expressed as increase fold. In C and D, LNCaP cells were left unchallenged or challenged with increasing concentrations of R1881 (from 0.1 to 10 nM) in the absence or presence of the Rh-2025u peptide. Cells were allowed to migrate (C) or invade (D) in Boyden's chambers and scored as described in "Methods" section. Data were expressed as increase fold. In A-D, Means and SEMs are shown. *n* represents the number of experiments. *p < 0.05 for the indicated experimental points versus the corresponding untreated cells.
